# Antecedents and consequences of silent treatment in close adult relationships: a systematic review

**DOI:** 10.3389/fpsyg.2026.1659694

**Published:** 2026-02-12

**Authors:** Avni Dubey, Ravinder Kumar, Akriti Srivastava, Rameshbabu Tamarana, Anupam Yadav, Vishal Sharma, Satvinder Singh Saini

**Affiliations:** 1 Department of Psychology, Central University of Karnataka, Gulbarga, India; 2 Department of Sports Psychology, Central University of Rajasthan, Ajmer, India; 3 Department of Psychology, Jagannath University, Bahadurgarh, India; 4 Department of Psychiatry, Post Graduate Institute of Medical Education and Research, Chandigarh, India

**Keywords:** close interpersonal relationships, communication avoidance, relationship satisfaction, silent treatment, social exclusion

## Abstract

**Background:**

The silent treatment, characterized by deliberate communication avoidance, is a common form of social exclusion that significantly impacts close adult relationships. Although, often regarded as a minor issue, its persistent use can lead to serious emotional and psychological consequences.

**Methods:**

This systematic review aimed to consolidate research findings to identify the antecedents and consequences of the silent treatment in interpersonal relationships. Following PRISMA (2020) guidelines, 15 peer reviewed articles were analyzed, and sourced from databases and search engines including PubMed, Google Scholar, Science Direct, and Web of Science.

**Results:**

The antecedents identified through these studies were emotional regulation, personality traits, manipulation tactics, cognitive processes, relational contexts and to some extent gender. The consequences identified for both givers and receivers of the silent treatment were decreased overall psychological health with long-term emotional distress and poor relationship satisfaction.

**Conclusion:**

These findings emphasize the need for improved communication strategies and interventions to address the detrimental effects of silent treatment in various relational contexts.

## Introduction

The “silent treatment” refers to a form of social exclusion or communication avoidance in which one person deliberately ignores or refuses to speak to another, often as a means of punishment or to express displeasure (Williams, 2007). Silent treatment is a relationship-specific form of ostracism (Zadro et al., 2008), that involves deliberate ignoring or exclusion to express disapproval or control within close relationships (Sommer et al., 2001). Unlike overt rejection, it often takes the form of “quiet silence,” where one partner emotionally withdraws and avoids interaction (Zadro et al., 2016). This quiet form of ostracism, shown through holding back, tuning out, or shutting down, reflects emotional disengagement rather than open conflict (Zadro, 2004). Unlike group-based ostracism aimed at punishment, silent treatment in adult relationships often serves to regulate emotions or assert personal boundaries (Gruter and Masters, 1986; Schachter, 1959). This behavior can manifest in various settings, including personal relationships, workplaces, and family dynamics, and its use is evident worldwide, across all cultures and types of relationships (Agarwal and Prakash, 2022). Buss et al. (1987) found that the silent treatment was one of the tactics individuals use to manipulate their partners in romantic relationships, while Falbo and Peplau (1980) identified it as a power tactic.

The conceptual picture of silent treatment can be understood with the help of several theoretical framework, the self-verification theory proposes that the people prefer the interaction confirming their self-view; perceived misrecognition can prompt withdrawal (Swann and Read, 1981; Agarwal and Prakash, 2022). The expectancy value theory holds that unmet expectations from the concerned person leads to less attraction and motivate corrective response and disengagement (Burgoon, 1993; Burgoon and Jones, 1976). Mind-Reading Expectations extend this logic - when a partner “should just know” one’s feelings, perceived neglect heightens disappointment and can elicit silence (Epstein and Eidelson, 1981; Wright and Roloff, 2015). Together, these perspectives explain silence as a response to threatened identity and unmet relational expectations, without over-specifying competing theories.

Furthermore, Smart Richman and Leary (2009) explain diverse responses to ostracism and social exclusion through the Multimotive Model of Rejection. This model proposes that experiences such as ostracism, rejection, and interpersonal withdrawal elicit similar immediate emotional reactions, including hurt feelings and lowered self-esteem. However, subsequent behavioral responses vary depending on individuals’ interpretations of the rejection experience rather than reflecting a single, uniform reaction to exclusion. According to the model, three competing motivational tendencies may be activated: efforts to restore social connection, impulses toward anger or retaliation, and tendencies to withdraw to avoid further rejection. Cognitive appraisals of the situation such as perceived fairness, expectations of relationship repair, the value placed on the relationship, and the perceived availability of alternative sources of belonging determine which motive becomes dominant. As the silent treatment is one form of ostracism (Sommer et al., 2001), it may function as an avoidant response aimed at protecting oneself from further emotional pain, a strategic attempt to prompt reconciliation, or a reaction rooted in anger when withdrawal is perceived as unjustified.

The experimental impact of silent treatment is substantial. Social exclusion activated the neural signal which activates physical pain, demonstrating its profound effect on social pain (Eisenberger et al., 2003) reduced psychological well-being, feeling of rejection and hurt emphasizing the detrimental effects on relational dynamics (Leary, 2003; Leary et al., 2006). Furthermore, it threatens the fundamental human needs for belonging, self-esteem, control, and meaningful existence (Williams, 2007, 2009). Receivers frequently report loneliness, and depression, and prolonged exposure may foster helplessness and disengagement (Baumeister and Leary, 1995; Cacioppo and Hawkley, 2003; Williams, 2001). Personality and affective variability further shape these dynamics; for instance, higher neuroticism relates to greater emotional instability, which may increase reliance on withdrawal strategies during conflict (Mader et al., 2023). However, the silent treatment is consequential form of interpersonal exclusion in close relationships. It reflects threatened identity and violated expectations, interacts with personality and emotion regulation, and varies by underlying motive.

### Rationale

The silent treatment can significantly influence emotional and psychological Wellbeing, potentially leading to stress, anxiety, and relationship deterioration for both parties involved (Gottman and Levenson, 2000; Pruitt and Carnevale, 1993). However, the underlying processes that shape this behavior have not been examined in detail, and its theoretical explanations remain limited (Williams et al., 1998). This systematic review aims to bring together the existing evidences on the antecedents and consequences of silent treatment, to clarify its mechanism and outcomes. By reviewing empirical studies and theoretical perspectives, this review seeks to provide a clearer understanding of how silent treatment emerges and its impact on individuals and relationships. Understanding these dynamics is crucial for developing interventions and improving interpersonal strategies, particularly in environments where silent treatment is prevalent.

## Methods

### Eligibility criteria

The eligibility criteria for this systematic review were established by following the PICO framework to ensure a comprehensive selection of relevant studies. The population (P) included studies that involved adult participants, aged 18 years and older, who were part of close interpersonal relationships such as romantic partners, spouses, family members, and friends. The intervention (I) focused on studies that specifically addressed the antecedents and consequences of silent treatment. The comparison (C) was not applicable, as this review did not require a direct comparison between interventions. The outcome (O) included studies that reported on the emotional, psychological, or relational impacts of silent treatment in close adult relationships.

Inclusion criteria required that the study design be qualitative, quantitative, or mixed-method, and the study must focus on the effects of silent treatment in interpersonal contexts. To ensure relevance and reliability, only studies published in English and with accessible full-text versions were included. Excluded were- unpublished manuscripts, conference abstracts, reviews, case studies, letters, and other types of gray literature, as these sources do not meet the methodological rigor required for systematic reviews. Additionally, studies not available in English or without accessible full texts were not considered.

### Information sources and search strategies

The studies for this systematic review were identified through multiple sources, including well-established academic databases: PubMed, Google Scholar, Science Direct, Web of Science, Psycnet, and Scopus. The search strategy utilized different search queries tailored to each database. In PubMed and Science Direct, the search was conducted using the term “Silent treatment” (Title/Abstract). For Web of Science, the search included the terms [TI = (“Silent treatment”)] OR AB = (“Silent treatment”). Psycnet employed the search term “Silent Treatment,” while in Scopus, the search was conducted using TITLE-ABS-KEY (“silent treatment”). Additionally, for Google Scholar, the manual search involved keywords such as silent treatment, interpersonal, close relationships, conflict, and communication.

### Selection process

The process was done in accordance with the PRISMA (Preferred Reporting Items for Systematic Reviews and Meta-Analyses; 2020) guidelines. The studies were collected and selected with the help of Zotero software. Duplicates were then removed. Title and abstract screening were conducted for the remaining articles. After that, screening was performed to eliminate the studies that were not accessible. Full-text screening was carried out for the remaining studies. Reasons for exclusion were recorded for all the excluded studies.

### Data collection process

The following data was extracted by the authors from the selected studies: the first author, publication year, research question, objectives, study design, sample size, sample characteristics, procedure, tools, data analysis, and results. Limitations of the individual studies were also extracted.

### Study risk of bias

The risk of bias in the studies was evaluated using the JBI checklist for cohort, qualitative, quasi-experimental, and analytical cross-sectional studies. The checklists used were the JBI critical evaluation tool for quasi-experimental investigations (Barker et al., 2024), for randomized controlled trials (Barker et al., 2023), for qualitative studies (Lockwood et al., 2015) and for analytical cross-sectional studies (Moola et al., 2020). There were four responses in these checklists: yes, no, unclear, or not applicable. One mark was given for the response “yes,” and zero for the rest of the responses. The final quality score for individual studies was determined through a standardization process in which, to obtain the standardized score, the score obtained in a checklist was divided by the maximum score and was then multiplied by 10. Based on these standardized scores, the studies were categorized into the following categories: strong (≥7), moderate (5–7), and weak (≤5).

### Study selection

**FIGURE 1:**
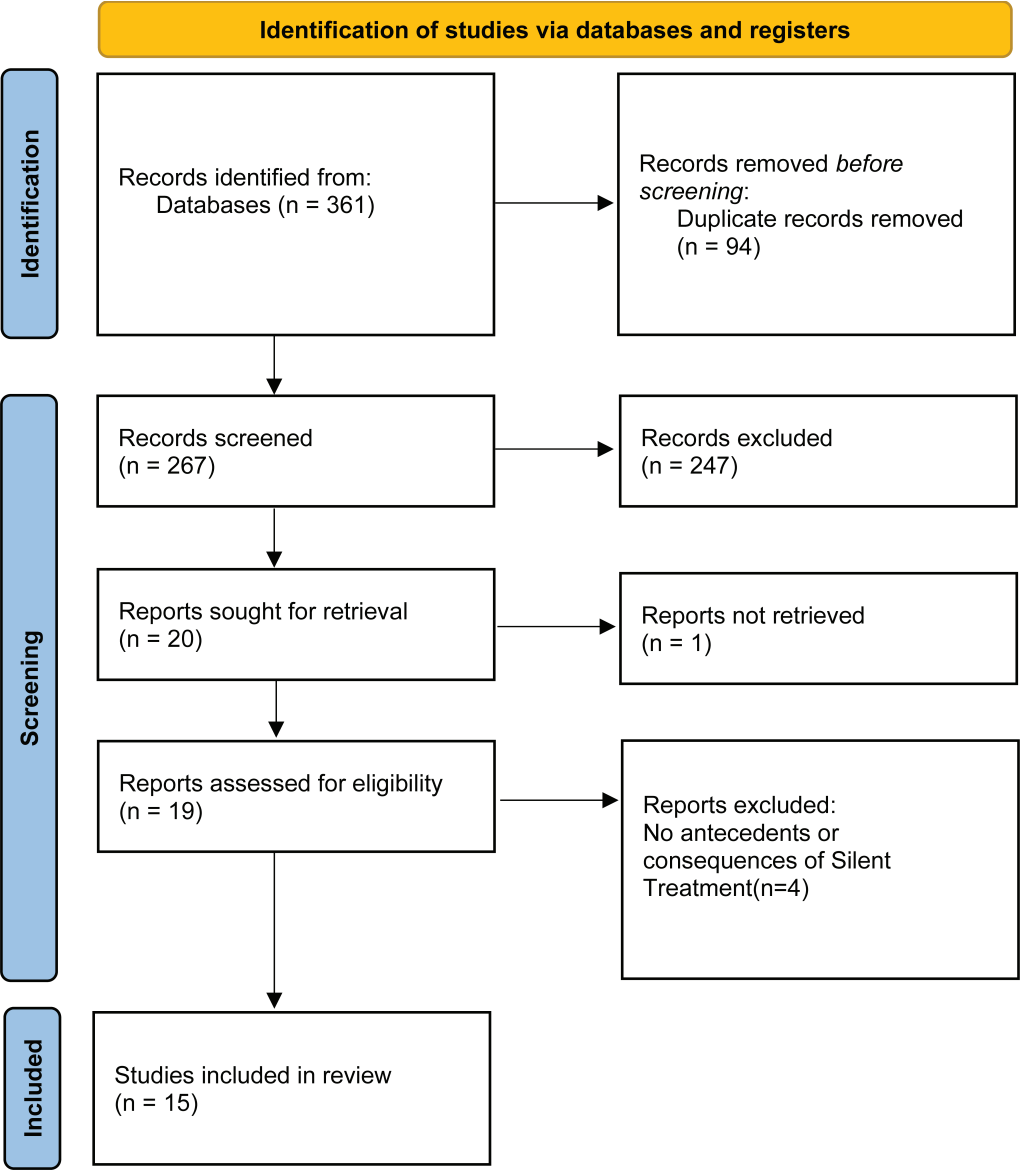
Showing PRISMA (2020) flow chart.

Using various databases, a total of 361 articles were identified through the specified search strategies and keywords. After removing 94 duplicates, the initial screening was conducted on the remaining 267 articles by reviewing their titles and abstracts. The inclusion criteria led to the selection of 20 articles for further examination, while 247 articles were excluded as their title and abstract didn’t align with the review focus. Specifically, most of the articles didn’t address silent treatment in close adult relationship. Among the 20 articles, the full text of one was unavailable, resulting in a review of 19 studies in total. Following the full-text review of these 19 articles, four were excluded because they did not adequately address the antecedents or consequences of the silent treatment. Hence, 15 studies were ultimately included in the review (Figure 1).

### Study characteristics

For the review, 15 studies were taken. The total number of participants was calculated to be 2,417 with the sample size ranging from small studies with 10 participants (Agarwal and Prakash, 2024), to larger studies with up to 411 participants (Liu and Roloff, 2015). The most common research design was Cross-sectional (*n* = 9), Quasi-Experimental (*n* = 4), and Qualitative (*n* = 3). The majority of the studies used self-reported data, while some also used Semi-structured Interviews and manipulated experimental conditions (Table 1).

**TABLE 1:** Showing study characteristics and findings.

S. no.	References	Research design	Methods	Results	Standardized JBI score
1	(Agarwal and Prakash, 2024)	Qualitative	Variables- emotional impact on source, relationship value, communication difficulties, attribution of silence, regret and resolution, relationship quality, post-silent treatment insights Tools- semi-structured interview Sample size- 15	Interviews revealed that giving the silent treatment created strong emotional strain, including anger, frustration, and guilt. Participants ended silence mainly to preserve valuable relationships but found communication difficult. When silence was seen as personal failure, self-esteem declined. Some used silence to terminate relationships and later regretted unresolved issues, while a few experienced improved understandings after discussion, showing silence can sometimes promote reflection if followed by dialogue. The study showed that silent treatment tends to harm emotional well-being and relationship quality, offering limited benefits unless followed by constructive communication.	7
2	(Agarwal and Prakash, 2022)	Qualitative	Variables- purpose of silent treatment, target emotions, threats to belongingness, impact on self-esteem, source perspective, negative emotional outcomes, self-esteem levels Tools- narrative method Sample size- 10	Silent treatment as emotionally taxing, often accompanied by frustration, hurt, and an internal struggle about whether to speak or withdraw. Many participants reported using silence either to avoid conflict or to signal distress when direct communication felt difficult, while others used it as a means of exerting control or creating emotional distance. The behavior was shaped by relationship values, individuals were more likely to break the silence when the relationship mattered, yet unresolved issues and regret commonly followed prolonged withdrawal. Overall, the findings showed that silent treatment is a complex coping strategy for the source, often driven by emotional overwhelm rather than intentional manipulation.	7
3	(Buss, 1992)	Cross sectional	Variables- agreeableness, coercion, silent treatment, manipulation tactics, giver’s personality, recipient’s personality, regression, behaviors (yelling, pouting, stony silence) Tools- self-reported tactics of manipulation, observer-reported tactics of manipulation, self-reported personality characteristics, spouse-observer reporting of personality characteristics, Interviewer-based observer reporting of personality characteristics Sample size- 214	Individuals low in agreeableness are more likely to use coercive tactics including the silent treatment as strategies to influence their partners, self-reported data of partners and the observation data both show strong associations between low agreeableness and withdrawal-based behaviors. The study also found that a spouse’s personality predicted manipulation tactics more strongly than the user’s own traits, meaning that partners low in agreeableness tended to evoke more regression, coercion, and silent treatment from the other person. Silent treatment frequently appeared in relationships marked by emotional volatility, where low agreeableness and higher neuroticism interacted to produce more controlling and distancing behaviors.	7.5
4	(Buss et al., 1987)	Cross sectional	Variables- silent treatment, manipulation tactics, self-other agreement, neuroticism, relationship issues, compatibility, power imbalances, dysfunctional relationship dynamics, emotional manipulation Tools- tactics of manipulation, eysenck personality questionnaire (EPQ), interpersonal adjective scales (IAS), interviewer judgments about couple relationships Sample size- 118	Silent treatment emerged as one of six primary manipulation tactics identified across romantic partners. Factor analyses across four behavioral measures confirmed six core manipulation tactics in close relationships, including Silent Treatment, Coercion, and Charm. Self-other agreement for overall manipulation during instigation was moderate (*r* = 0.44, *p* < 0.001), while agreement for Silent Treatment during conflict termination was weaker (*r* = 0.29, *p* < 0.05). Individuals higher in neurotic tendencies reported greater use of Silent Treatment (r ≈.33, *p* < 0.001) and Regression (*r* = 0.28, *p* < 0.01) when influence attempts occurred. Relationship similarity showed a negative link with manipulation, with self-reported total manipulation scores correlating *r* = −0.31. Mean tactic scores demonstrated that negative strategies such as Silent Treatment were used reliably across situations. Together, the results indicate stable individual differences, where those higher in emotional instability rely more on subtle withdrawal tactics to control close others.	7.5
5	(Cataldi and Reardon, 1996)	Cross sectional	Variables- interpersonal orientation (IO), manipulation tactics, gender differences, silent treatment Tools- interpersonal orientation scale, manipulation tactics index, Demographic questionnaire, subjective closeness index Sample size- 172	Women with higher interpersonal orientation reported using manipulation tactics, including indirect strategies such as the silent treatment, more often than women low in interpersonal orientation, whereas men showed no such differences across interpersonal-orientation levels. Overall, neither gender nor interpersonal orientation alone adequately explained manipulation-tactic use, as both factors interacted with relational context rather than consistently predicting behavior. The researchers also found no meaningful gender differences in the overall frequency of manipulation tactics, indicating that men and women relied on these behaviors at comparable rates. Together, the results suggest that interpersonal orientation shapes influence-tactic use primarily among women, while gender itself is not a reliable predictor of manipulation patterns in close relationships.	7.5
6	(Hyla, 2015)	Cross sectional	Variables- use of silent treatment, overall relationship satisfaction, levels of meanness (in men), disinhibition (in both genders) Tools- TriPM, triarchic psychopathy measure; KDM-2, relationship satisfaction scale, KWS, influence tactics questionnaire Sample size- 131	Higher meanness in men significantly predicted greater use of the silent treatment and other hard influence tactics in romantic relationships (β = 0.41, *p* < 0.01). Dis-inhibition in both men and women was also a significant predictor of silence and withdrawal (β = 0.36, *p* < 0.01). Meanness showed a negative association with relationship satisfaction (β = −0.32, *p* < 0.05), indicating that partners high in callousness tended to report poorer relational wellbeing. Overall, the triarchic traits especially meanness and disinhibition were linked to more manipulative, impulsive, and relationally harmful strategies.	7.5
7	(Liu and Roloff, 2015)	Cross sectional	Variables- use of silent treatment, use of stonewalling, emotional exhaustion, level of rumination, cognitive reappraisal (short-term vs. long-term effects) Tools- rumination scale, cognitive reappraisal scale, silent treatment scale, stonewalling scale, emotional exhaustion scale Sample size- 411	Longer durations of complaint-withholding were positively related to irritant frequency (*B* = 0.232, 95% CI [0.105, 0.358]) and in turn to irritant importance (*B* = 0.565, 95% CI [0.476, 0.653]). Greater irritant importance predicted higher emotional distress from withholding (B = 0.434, 95% CI [0.351, 0.518]), and emotional distress significantly predicted regret for withholding (*B* = 0.336, 95% CI [0.257, 0.416]). Although relational intimacy declined with longer withholding (*B* = 0.279, 95% CI [0.163, 0.395]), intimacy did not significantly mediate the path to regret.	7.5
8	(Rittenour et al., 2019)	Cross sectional	Variables-use of silent treatment, relational satisfaction, self-esteem, tolerance for disagreement, parent’s use of silent treatment Tools- modified version of grievance expression scale, tolerance for disagreement scale, self-esteem scale Sample size- 182	Silent treatment found positively associated with adult children’s own use of silent treatment (*B* = 0.32, *p* < 0.01). Adult children’s self-esteem was negatively linked to their silent treatment use when experiencing anger (*B* = −0.42, *p* < 0.01). Parent admission of displeasure predicted higher relational satisfaction with the parent (*B* = 0.20, *p* < 0.05) and greater feelings of control by the adult child (*B* = 0.17, *p* < 0.05). No significant mediation by parent identification was found in the path from parent to child silent-treatment behaviors (ab = −0.001, 95% CI [−0.05, 0.05]). Thus, silence appears socially transmitted and inversely related to self-esteem, emphasizing communication modeling within families.	7.5
9	(Mandal and Wierzchoń, 2019)	Cross sectional	Variables- use of silent treatment, level of shyness, sex differences. Tools- self-presentation questionnaire, attitude toward adonization – scale A, influence in close relationships between women and men questionnaire, revised cheek and buss shyness scale (RCBS) Sample size- 234	Shy individuals reported significantly lower use of self-promotion (*M* = 41.80) and higher use of self-deprecation compared to their less shy counterparts (*M* = 46.14, *p* < 0.001). Higher shyness also corresponded with reduced use of adonization in close relationships (*M* = 57.11 vs. 70.54, *p* = 0.004). Regression results confirmed shyness as a significant predictor of self-depreciation [F (1,231) = 37.76, *p* < 0.001] and self-promotion [F (1,231) = 8.41, *p* < 0.001]. Additionally, shyness predicted greater reliance on hard influence tactics, including silent treatment, with a significant model explaining 5.7% of its variance [F (1,231) = 8.02, *p* < 0.001]. Additionally, shyness predicted greater reliance on hard influence tactics, including silent treatment, with a significant model explaining 5.7% of its variance [F (1,231) = 8.02, *p* < 0.001].	7.5
10	(Sommer et al., 2001)	Quasi-experimental	Variables- performance on self-regulatory task, type of interaction (conversing vs. ignoring), desirability of relationship partner (likeable vs. unlikeable) Tools- social attitudes questionnaire (SAQ), first impressions questionnaire (FIQ), criminal judgment task, symbol matching task Sample size- 297	Across both studies, causally unclear ostracism consistently produced stronger threats to belongingness and self-esteem than clear ostracism (e.g., χ^2^ values around 3.7–7.1, *p* ≤ 0.05), and oblivious ostracism elicited even greater belongingness threat than intentional forms. Sources repeatedly described ostracism as effective and controlling, while targets more often reported withdrawing, reciprocating the silence, and feeling resentful. Targets also reported a wider range of negative emotions including frustration, guilt, and loneliness whereas, sources mainly noted anger. Together, the studies show that silent treatment harms targets more intensely, while sources tend to perceive it as a justified and goal-achieving strategy	6.25
11	(Sommer and Yoon, 2013)	Quasi-experimental	Variables- self-regulatory task performance, type of interaction (silent treatment vs. conversation), Desirability of relationship partner (likeable vs. unlikeable) Tools-social attitudes questionnaire (SAQ), first impressions questionnaire (FIQ), criminal judgment task, recognition memory task Sample size- 120	Participants who conversed with a highly unlikeable person performed worse on a self-regulation task than those who ignored that person [F (1, 66) = 4.52, *p* = 0.037]. Conversely, when dealing with a highly likeable individual, those who conversed performed slightly better than those who ignored them [F (1, 66) = 3.91, *p* = 0.052]. Study 2 replicated this crossover interaction using a different self-regulation measure [F (1, 87) = 5.12, *p* = 0.026], supporting the idea that silence may help conserve regulatory resources during unpleasant social interactions.	6.25
12	(Williams et al., 1998)	Qualitative	Variables- Threatened needs (belonging, self-esteem, control, meaning), perceived belonging (source’s perspective), perceived control (source’s experience), use of silent treatment, recipient’s experience (avoidance and lack of communication) Tools- subjective listing of behavior and feeling when giving and receiving the silent treatment Sample size- 109	Receivers of the silent treatment reported significantly greater threats to belongingness (*t* = −4.05, *p* < 0.001) and meaningful existence (*t* = −3.48, *p* = 0.001) compared with non-recipients. They also reported lower self-esteem (*t* = −2.76, *p* = 0.007) and reduced sense of control (*t* = −2.43, *p* = 0.016) when subjected to silent treatment. Those administering the silent treatment reported greater perceived control (*t* = 2.65, *p* = 0.009) than those not using it. Overall, the findings indicate that the silent treatment functions as a powerful relational tool that damages core psychological needs for the recipient while elevating feelings of power for the sender.	7
13	(Wirth et al., 2010)	Quasi-experimental	Variables- feelings of exclusion and neglect, satisfaction of basic needs (belonging, self-esteem, meaningful existence), mood, relational evaluation, perception of partner’s traits (positive and negative), aggressive tendencies, prosocial behavior, type of gaze (averted vs. direct) Tools- Movies of actors’ faces (direct gaze and averted gaze), Visualization task (using male or female computer avatar) Sample size- 188	Participants exposed to averted eye gaze reported significantly higher feelings of exclusion [t (23) = 2.99, *p* = 0.006, *d* = 1.17] and being ignored [t (23) = 5.22, *p* < 0.001, *d* = 2.09] compared to those exposed to direct eye gaze. They also showed lower basic-need satisfaction [t (23) = −2.26, *p* = 0.03, d = 0.89] and self-esteem [t (23) = −2.40, *p* = 0.03, d = 0.94]. In a larger sample, averted gaze led to reduced relational evaluation [t (107) = −3.96, *p* < 0.001, d = 0.76], lower basic-need fulfillment [t (131) = −4.06, *p* < 0.001, d = 0.70], and increased aggressive behavioral temptations [t (131) = 2.23, *p* = 0.03, d = 0.39]. Lastly, in Study 3 implicit self-esteem was lower after averted gaze (*M* = 141.7 ms) vs. direct gaze (*M* = 298.9 ms), t (27) = −2.29, *p* = 0.03, d = 0.85.	6.25
14	(Wright and Roloff, 2009)	Cross sectional	Variables- use of silent treatment, use of stonewalling, admission of being upset, relational commitment, gender Tools- self-report questionnaires on relationship commitment, grievance expression, and silent treatment Sample Size- 108	Relational commitment was found negatively associated with self-reported use of the silent treatment when a partner asked if there was a problem (β = −0.24, *p* < 0.01). Further, commitment also inversely predicted stonewalling behavior (β = −0.22, *p* < 0.05), indicating that more committed partners were less likely to withdraw communication entirely. Gender did not significantly moderate these effects, though women reported higher use of silent treatment overall [t (106) = 2.10, *p* = 0.04]. These findings suggest that higher relational commitment relates to fewer avoidance-based responses during relational conflict.	10
15	(Wright and Roloff, 2015)	Cross sectional	Variables- use of silent treatment, feelings of upset, emotional neglect, partner’s emotional awareness Tools- relationship belief inventory, upset scale, silent treatment scale, combativeness scale, relational satisfaction scale Sample size- 108	Individuals with higher mind-reading expectations (MRE) felt more upset when partners failed to recognize their emotions, and this upset predicted greater combative behavior (indirect effect = 0.32, 95% CI [0.16, 0.55]). MRE also predicted increased use of silent treatment through heightened upset (indirect effect = 0.45, 95% CI [0.21, 0.79]). In both cases, upset fully mediated the associations, as the direct paths from MRE to combative behavior and silent treatment became non-significant. These findings show that emotional upset not MRE alone drives withdrawal and confrontational responses to relational problems.	10

### Risk of bias in studies

In order to evaluate the quality of the final studies included, the JBI quality appraisal criteria were used. Through the interpretation based on the standardized JBI scores, 12 studies were identified to have a high quality (less risk of bias) and three studies had moderate quality (medium risk of bias). The reason that most of the studies had less risk of bias was the clear description of the inclusion and exclusion criteria, subjects and the study setting, and the measurement of the outcomes using valid and reliable ways. A common reason of moderate quality was not mentioning the confounding variables.

## Results

The aim of this Systematic Review was to get a clear and conceptual understanding of silent treatment in close adult interpersonal relationships by identifying its antecedents and consequences. With the review of 15 articles (Table 1), the antecedents and the consequences that were identified for the givers and receivers of Silent Treatment are discussed below.

Emotional regulation found as key reason of emotional regulation. Silence was frequently used by the individuals to manage distressing emotions or to regain control during conflict (Agarwal and Prakash, 2022). Qualitative data revealed that givers described silence as a self-protective response to emotional overload, helping them avoid escalation but often creating additional tension (Agarwal and Prakash, 2024). Individual high on neuroticism was more likely to engage in silence, reflecting its connection with emotional volatility (Buss et al., 1987). The silent treatment, therefore, functions as an emotion-focused coping mechanism, offering short-term regulation but compromising relational intimacy over time.

Buss (1992) found that individuals with low agreeableness relied on coercive and withdrawn tactics, including the silent treatment, to influence partners’ behavior. Similarly, high neuroticism was linked with emotional instability and a greater likelihood of employing withdrawal strategies during relational conflict (Buss et al., 1987). Research also suggests that low self-esteem increases both the use and receipt of silent treatment, implying a cyclical dynamic in which insecure individuals perpetuate avoidance behaviors (Agarwal and Prakash, 2022; Rittenour et al., 2019). Mandal and Wierzchoń (2019) extended these findings by showing that shyness and social inhibition predict indirect conflict behaviors like sulking and silence particularly among women.

Researches revealed silent treatment as interpersonal manipulation tactics for punishing partners’ behavior. Buss et al. (1987) classified silent treatment among six manipulation tactics used to alter partners’ behavior without overt confrontation. Similarly, Cataldi and Reardon (1996) found women high on interpersonal orientation are more likely to use silence to elicit compliance or express dissatisfaction while minimizing overt conflict. This interpretation aligns with social exchange and power theories, which propose that silence can serve as a low-cost, high-impact means of control within asymmetric relationships. Hyla (2015) further observed that psychopathy linked traits such as meanness and disinhibition increase the likelihood of using silence as a manipulative tactic, indicating that moral disengagement and impulsivity intensify this behavior.

Cognitive factors also appear central to understanding the onset and persistence of silence. Liu and Roloff (2015) demonstrated that rumination and repetitive negative thinking about conflict was strongly associated with the use of both silent treatment and stonewalling. Individuals who over analyzed transgressions were more likely to withdraw communication, perpetuating emotional distance. Interestingly, cognitive reappraisal initially increased silence, perhaps as a deliberate delay to calm emotions, but reduced it over time as individuals reframed the conflict. These findings highlight the dual role of cognition in either exacerbating or mitigating silent behavior, depending on whether thought patterns promote emotional insight or fixation.

Wright and Roloff (2015) found that partners often employed the silent treatment when they perceived emotional neglect or low empathy from their counterparts. In such cases, silence functioned as both protest and punishment expressing unmet emotional needs. Couples with low compatibility or poor communication skills tended to use silence more frequently (Buss et al., 1987), reinforcing a pattern of withdrawal and distance. In contrast, greater relational commitment predicted lower use of silence, suggesting that invested partners prefer repair-oriented strategies (Wright and Roloff, 2009). These findings indicate that the silent treatment is not only a product of personal disposition but also a reflection of the relational climate, where unmet needs, emotional neglect, and perceived inequality interact to sustain avoidance.

Evidence of showing silent treatment among the gender found mixed but significant. Women generally reported higher use of the silent treatment to communicate dissatisfaction or exert influence, while men were more likely to use overt confrontation or emotional distancing (Wright and Roloff, 2009; Cataldi and Reardon, 1996). Hyla (2015) observed that men high in meanness showed greater use of silence, correlating negatively with relationship satisfaction. Meanwhile, Mandal and Wierzchoń (2019) found that shy women tended to adopt non-verbal avoidance tactics such as sulking and silence, reflecting gendered socialization toward indirect conflict management. Collectively, these studies imply that gender moderates the function rather than the frequency of silent treatment where women tend to use it communicatively, whereas men use it defensively or as a dominance assertion. People receiving the silent treatment often feel ignored, undervalued, and experience threats to belongingness and self-esteem (Williams et al., 1998). In Agarwal and Prakash (2024), qualitative findings revealed that while some individuals gained temporary clarity or reflection after silence, most experienced relational strain and regret. Silent treatment can serve as a maladaptive response to emotional distress, emphasizing the need for better communication (Wright and Roloff, 2015).

Emotional consequences are extensive for both givers and receivers. Receivers frequently report loneliness, guilt, sadness, and reduced self-worth (Agarwal and Prakash, 2022), while givers experience anger, frustration, and emotional exhaustion when silence prolongs beyond intent (Agarwal and Prakash, 2024). Rittenour et al. (2019) observed that repeated exposure to parental silence predicted lower self-esteem and relational satisfaction among adult children, highlighting intergenerational transmission effects. Wirth et al. (2010) extended this by demonstrating that non-verbal cues such as averted gaze trigger the same exclusionary pain as verbal silence, reducing mood and increasing aggression. These findings underscore that the silent treatment elicits both social pain and physiological stress comparable to other forms of rejection.

Experimental study done by Sommer et al. (2001), Sommer and Yoon (2013) connected silent treatment to cognitive resource depletion. Ignoring or being ignored by a likeable partner impaired self-regulatory performance, while avoiding an unlikeable person preserved mental energy. These results indicate that the cognitive cost of silence depends on the social desirability of the partner, suggesting adaptive and maladaptive uses of withdrawal. Over time, habitual silence may reinforce avoidance tendencies, reducing problem-solving ability and empathy within relationships. Several studies highlight that chronic reliance on silence contributes to long-term emotional fatigue and relationship erosion. Agarwal and Prakash (2022) found that individuals often regretted prolonged silence but struggled to initiate reconciliation due to shame and fear of rejection. Liu and Roloff (2015) reported that rumination during silent episodes predicted emotional exhaustion and relational disengagement, creating self-perpetuating cycles of withdrawal. Cumulatively, these effects erode relationship satisfaction and psychological wellbeing, emphasizing the need for intervention strategies that foster open dialogue and emotional literacy.

## Discussion

The systematic review has provided a clear and comprehensive summary of the available evidence about the antecedent and consequence of the silent treatment. Finding across fifteen studies reveal that silence operates as both coping strategy and an interpersonal control mechanism. The broader picture of giving the silent treatment can be understood by these findings- (i) difficulty with regulation of emotions, (ii) manipulation or power management tactic and (iii) avoidance of negative relational experiences. Gender and personality dispositions can play the significant moderator on the basis of available sociocultural factors. Consequently, these factors are influencing the overall the psychological health (i.e., psychological, emotional, cognitive, and behavioral) of an individual in the long term social relational outcomes. Overall synthesis of the reviewed studies suggests that silent treatment is not a single-purpose response, but a multi-determined interpersonal behavior emerging from the interaction of individual vulnerabilities and relational conditions.

Many individuals use silent treatment as a way to manage their negative emotions as a response to emotional distress (Agarwal and Prakash, 2022; Buss et al., 1987; Wright and Roloff, 2015). When individuals feel that they are misunderstood or misjudged, they experience discomfort. To protect their self-view, they may withdraw from the interaction. Agarwal and Prakash (2022) explained this by suggesting that when individuals feel that their identity or feelings are not recognized by others, they might use the silent treatment to maintain their sense of self and emotional balance. Such behavior aligns with self-verification processes, whereby individuals disengage from interactions that threaten their perceived identity. However, while silence may temporarily regulate emotion, it also prevents the repair of relational ruptures, leading to enduring dissatisfaction. Across different designs and samples, the findings consistently show that silence may lessen immediate emotional arousal for the giver but, over time, erodes intimacy and leaves underlying issues unresolved, creating a paradox of short-term relief and long-term relational cost.

When used as a manipulation tactic, the silent treatment is used to either control the situation or to terminate unwanted behavior (Buss et al., 1987; Cataldi and Reardon, 1996). Expectancy Violation Theory (Burgoon, 1993) and Mind-Reading Expectations (Epstein and Eidelson, 1981) provide theoretical support, suggesting that individuals resort to silence when relational expectations are breached or empathy is lacking. In this sense, silence becomes a communicative signal of disapproval and an effort to restore perceived relational order. Wright and Roloff (2015) found that such unmet expectations can lead individuals to use the silent treatment as a way of expressing dissatisfaction or to punish the partner for failing to meet these unspoken needs. Negative situations that often trigger the use of the silent treatment include feelings of emotional neglect from a partner, having an unresolved grievance, or avoiding confrontation (Wright and Roloff, 2015; Wright and Zimmermann, 2019). Partners may perceive conversation as risky and choose silence as a safer, though ultimately counterproductive, response. This behavior illustrates avoidance-oriented coping in which short-term conflict reduction substitutes for authentic engagement. Taken together, the included studies indicate that silence functions as “communication by withdrawal,” where the lack of response itself becomes a message used to influence, protest, or punish when direct dialogue feels too threatening or unproductive.

Personality factors strongly predict the likelihood of using the silent treatment. Low agreeableness and high neuroticism consistently correlate with its use (Buss, 1992; Buss et al., 1987), confirming links between emotional instability and withdrawal behavior (Mader et al., 2023). Low self-esteem also contributes, as individuals lacking confidence in their worth both employ and receive silence more often (Agarwal and Prakash, 2022; Rittenour et al., 2019). Gender moderates these tendencies- women typically adopt silence to express grievances or de-escalate conflict, reflecting socialization toward relational maintenance (Wright and Zimmermann, 2019; Vigil, 2009). Conversely, men high in meanness or psychopathy-related traits use silence coercively, linking it to dominance and reduced satisfaction (Hyla, 2015). Collectively, these findings highlight that the antecedents of silence blend personality, emotional vulnerability, and situational context. This pattern suggests that silent treatment is not random or purely situational; rather, it reflects relatively stable dispositional tendencies that shape how individuals cope with threat, anger, and unmet needs in close relationships.

The consequences of silent treatment significantly impact both the individual using it and the recipient. The receivers of silent treatment frequently report feeling ignored, undervalued, guilty and lonely (Agarwal and Prakash, 2022). They experience feelings of exclusion and neglect, and reduced satisfaction with their basic human need, especially feeling a threat to their need for belongingness (Wirth et al., 2010; Agarwal and Prakash, 2022). They experience reduced self-esteem, leading to decreased relationship satisfaction (Buss et al., 1987; Hyla, 2015; Rittenour et al., 2019). While it may provide temporary emotional relief for the giver of the silent treatment, the long-term effects tend to be detrimental, fostering relational deterioration (Wright and Roloff, 2015). However, when followed by constructive dialogue, silent treatment can sometimes yield deeper insights and facilitate relationship growth (Agarwal and Prakash, 2024). The reviewed evidence therefore suggests that the eventual transition from silence back to dialogue is critical: when withdrawal is followed by repair-oriented communication, it may facilitate reflection, but when it remains unresolved, it consolidates disconnection and chronic dissatisfaction. The giver may also face different consequences based on how much they like the receiver, because while giving the silent treatment to an unlikeable can help them save their self-regulatory resources, giving it to a likeable person can deplete the giver of their cognitive and physical performance by making them give up much more easily (Ciarocco et al., 2001).

Long-term consequences of silent treatment show a cycle of emotional exhaustion and unresolved issues, affecting both individual wellbeing of both, the target and the source, and relational dynamics (Rittenour et al., 2019; Liu and Roloff, 2015). This explains why James (1890) considered it to be worse than the harshest form of punishment. Silent treatment generally has negative effects on individuals. However, it is important to view it from both the giver’s and the receiver’s perspectives. Theoretically, (Burgoon, 1993; Burgoon and Jones, 1976) some people may use silent treatment to distance themselves from unpleasant or stressful situations, which might serve a self-protective purpose. From the receiver’s side, though, its meaning and impact are often unclear, leading to emotional hurt and negative consequences.

Zadro et al. (2008) demonstrated that ostracism involving a romantic partner has particularly silent interpersonal meaning, as partners are typically expected to meet core needs such as belonging and validation. This indicated that partner based withdrawal can prompt an individual to reassess the relationship itself. People who gave silent treatment shows the showed decreased performance on a physical stamina task and they gave up more quickly on unsolvable puzzles compared to people who didn’t give silent treatment (Ciarocco et al., 2001). Across the studies included, chronic or repeated use of silence appears to shift from a short-term coping response to a maladaptive interpersonal pattern that undermines psychological safety, reduces problem-solving, and weakens long-term relationship stability.

## Limitation and future directions

This systematic review provides valuable insights into the antecedents and consequences of the silent treatment in close relationships, still there are several limitations that must be acknowledged. First, the majority of the studies included in the review rely on self-reported data, which can be prone to biases such as social desirability or inaccurate recollections. The studies included in the review mostly associated with romantic or marital relationships, which limits the understanding of silent treatment in close relationships. Additionally, there was limited research on the receiver’s traits and how they influence the likelihood of receiving silent treatment, which could be an important factor in understanding the dynamics of this behavior. Furthermore, the review synthesizes findings from only 15 studies, and this relatively small body of research limits the conclusions about the phenomenon. Although the studies examined the emotional and psychological consequences through qualitatively and cross-sectional researches. But there is a need for more longitudinal research to explore the long-term effects of the silent treatment on relationships and individual well-being. More empirical researches are required to understand the impact of different types of silent treatment (e.g., passive-aggressive vs. temporary withdrawal) and whether they produce different outcomes in relationships. More diverse and representative samples, including from different cultures, could further enhance the understanding of how silent treatment functions in different relational dynamics.

## Implications

The findings of the review highlight that the silent treatment is a multi-determined emotional response that can be interpreted as an outcome of individuals’ motives for using it. From a clinical and counseling perspective, it can be addressed as part of therapeutic intervention in relationship dynamics. The review showed an association between silent treatment and loneliness, emotional exhaustion, and long-term relationship dissatisfaction. Practitioners can assess the frequency and causes of silence in the relationship, and interventions can be provided to enhance emotional awareness, communication skills, and conflict resolution techniques. This may help individuals replace withdrawal with more adaptive strategies. Importantly, silence may not always be harmful when used for emotional regulation, but prolonged and unresolved silence can have negative impacts on relationship dynamics in the long term.

Therefore, therapeutic efforts should focus on facilitating the transition from silence to constructive dialogue rather than simply discouraging withdrawal altogether. Evidence from the review also highlighted gendered patterns, where women tend to use silence communicatively, while men may use it defensively or coercively. This suggests that interventions should be sensitive to gendered expectations around emotional expression and communication, rather than adopting a one-size-fits-all approach.

Finally, the review underscores the broader relational and psychological costs of chronic silent treatment. For relationships, persistent silence can normalize avoidance and disengagement, increasing the risk of long-term instability. These implications emphasize the importance of addressing silent treatment not only as an individual behavior but as a relational pattern, which requires mutual awareness and repair-oriented responses.

## Conclusion

In conclusion, the silent treatment is a complex behavior with both emotional and relational consequences for those who give and receive it. The antecedents of this behavior include emotional regulation issues, manipulation tactics and power tactics, and reactions towards the negative or unenviable conditions of relationship, often linked to personality traits such as neuroticism and low agreeableness. While the silent treatment can temporarily relieve emotional distress for the giver. It tends to have long-term detrimental effects, including feelings of isolation, low self-esteem, and decreased relationship satisfaction for the receiver. Although there are instances where constructive dialogue after the silent treatment can lead to positive outcomes. The overall impact of this behavior is generally negative, contributing to relational deterioration over time. Given the psychological and emotional toll, it takes on both individuals involved. Addressing the underlying causes and improving communication strategies in relationships is essential for reducing the harmful effects of the silent treatment. Future research should aim to explore more diverse populations with more experimental understanding form both receiver and givers perspective.

## Data Availability

The original contributions presented in this study are included in this article/supplementary material, further inquiries can be directed to the corresponding author.
